# French Experience of 2009 A/H1N1v Influenza in Pregnant Women

**DOI:** 10.1371/journal.pone.0013112

**Published:** 2010-10-05

**Authors:** Grégory Dubar, Elie Azria, Antoine Tesnière, Hervé Dupont, Camille Le Ray, Thomas Baugnon, Sophie Matheron, Dominique Luton, Jean-Christophe Richard, Odile Launay, Vassilis Tsatsaris, François Goffinet, Alexandre Mignon, for the French Registry on 2009 A/H1N1v during pregnancy

**Affiliations:** 1 Département d'Anesthésie-Réanimation, Hôpital Cochin, Assistance Publique - Hôpitaux de Paris, Université Paris Descartes, Paris, France; 2 Service de Gynécologie-Obstétrique, Hôpital Bichat, Assistance Publique - Hôpitaux de Paris, Université Paris Diderot, Paris, France; 3 Département d'Anesthésie-Réanimation, Hôpital d'Amiens, Université d'Amiens, Amiens, France; 4 Service de Gynécologie-Obstétrique, Hôpital Cochin, Assistance Publique - Hôpitaux de Paris, Université Paris Descartes, Paris, France; 5 Département d'Anesthésie-Réanimation, Hôpital Necker, Assistance Publique - Hôpitaux de Paris, Université Paris Descartes, Paris, France; 6 Service de Maladies Infectieuses et Tropicales, Hôpital Bichat, Assistance Publique - Hôpitaux de Paris, Université Paris Diderot, Paris, France; 7 Service de Gynécologie-Obstétrique, Hôpital Beaujon, Assistance Publique - Hôpitaux de Paris, Université Paris Diderot, Paris, France; 8 Service de Réanimation Médicale, Hôpital Charles Nicolle, Université de Rouen, Rouen, France; 9 Centre d'Investigation Clinique de Vaccinologie, Hôpital Cochin, Assistance Publique - Hôpitaux de Paris, Université Paris Descartes, Paris, France; University of Texas Medical Branch, United States of America

## Abstract

**Background:**

The first reports on the pandemic influenza 2009 A/H1N1v from the USA, Mexico, and Australia indicated that this disease was associated with a high mortality in pregnant women. The aim of this study was to describe and compare the characteristics of severe critically ill and non-severe pregnant women with 2009 A/H1N1v-related illness in France.

**Methodology/Principal Findings:**

A national registry was created to screen pregnant women with laboratory-confirmed 2009 A/H1N1v influenza. Three hundred and fifteen patients from 46 French hospitals were included: 40 patients were admitted to intensive care units (severe outcomes), 111 were hospitalized in obstetric or medical wards (moderate outcomes), and 164 were outpatients (mild outcomes). The 2009 A/H1N1v influenza illness occurred during all pregnancy trimesters, but most women (54%), notably the severe patients (70%), were in the third trimester. Among the severe patients, twenty (50%) underwent mechanical ventilation, and eleven (28%) were treated with extracorporeal membrane oxygenation. Three women died from A/H1N1v influenza. We found a strong association between the development of a severe outcome and both co-existing illnesses (adjusted odds ratio [OR], 5.1; 95% confidence interval [CI], 2.2–11.8) and a delay in oseltamivir treatment after the onset of symptoms (>3 or 5 days) (adjusted OR, 4.8; 95% CI, 1.9–12.1 and 61.2, 95% CI; 14.4–261.3, respectively). Among the 140 deliveries after 22 weeks of gestation known to date, 19 neonates (14%) were admitted to a neonatal intensive care unit, mainly for preterm delivery, and two neonates died. None of these neonates developed 2009 A/H1N1v infection.

**Conclusions:**

This series confirms the high incidence of complications in pregnant women infected with pandemic A/H1N1v observed in other countries but depicts a lower overall maternal and neonatal mortality and morbidity than indicated in the USA or Australia. Moreover, our data demonstrate the benefit of early oseltamivir treatment in this specific population.

## Introduction

Data from previous influenza pandemics have indicated that pregnant women have a higher risk for morbidity and mortality than do non-pregnant women [1]–[4]. The emergence of pandemic influenza A/H1N1 in America and Australia in early 2009 raised further awareness and concern in Europe [5], [6]. Indeed, the first reports from the Centers for Disease Control (CDC) [7] and Australia [8] indicated a severe mortality and even susceptibility to an increase in the 2009 maternal mortality ratio in the United States [9].

The A/H1N1v pandemic was declared by the World Health Organization on June 11^th^, 2009. Alerts about the increased mortality in pregnant women arrived from the Centers for Disease Control (CDC) in late June [7]. The “Collège National des Gynéco-Obstétriciens Français” (CNGOF), the “Société Française d'Anesthésie Réanimation” (SFAR), the “Société de Pathologie Infectieuse de Langue Française” (SPILF), and the French Ministry of Health published guidelines in August 2009 on the management of influenza-like illness (ILI) in pregnant women, which were actualized in November 2009 [10]–[11].

Briefly, whenever a pregnant woman presented with possible ILI, *i.e.,* fever >37.8°C and cough or sore throat, clinicians were asked to assess the illness severity and respiratory compromise using physical exams and tests, such as pulse oximetry, chest X-ray or arterial blood gases, as clinically indicated. Clinical and social risk factors (*e.g.*, coexisting diseases such as asthma, obstetric issues such as preterm labor, and inability to perform self-care or to arrange follow-up if necessary) were also assessed. In cases of severe disease, admission to an ICU was recommended, whereas women with mild or moderate diseases were referred to obstetric or medical wards to exclude other possible diagnoses, to perform an obstetric follow-up, and to initiate treatment. Women with mild outcomes without clinical or social risk factors were treated as soon as possible in an ambulatory setting. Antiviral treatment (oseltamivir: 75 mg *per os* twice per day for 5 days) was initiated as soon as possible after the onset of symptoms. Systematic reverse transcriptase-polymerase chain reaction (RT-PCR) for influenza A/H1N1 using nasal or throat swabs was performed during the pandemic period (after December 8, it was only recommended for severe patients), whereas it was discontinued for the general population. Pregnant women were identified as one of the initial target groups to receive the 2009 A/H1N1v monovalent non-adjuvant vaccine as soon as it was available (November 20^th^, 2009 in France).

Meanwhile, the National Institute for Public Health Surveillance (InVS, Institut National de Veille Sanitaire) recorded all cases of pandemic flu, either confirmed or suspected and either severe or moderate, in the French general population until November 2, at which time surveillance was only maintained for the most severe patients (those requiring intensive care) [12]. Therefore, no data are available regarding the number of pregnant or postpartum women with confirmed or suspected H1N1 pandemic flu (hospitalized or not), except for the most severe patients who were hospitalized in the ICU in France. Moreover, the InVS registry could not address the identification of risk factors involved in the development of the most severe outcomes of flu among pregnant women or the obstetric or neonatal outcomes.

We launched a web-based national registry (R3G, French National Registry on Flu during Pregnancy) under the aegis of four medical societies (CNGOF, SFAR, SRLF, and SPILF) and a collaboration with REVA (a specific French registry on critical care and ventilation). The aim of this registry was to record cases of pregnant or postpartum women with laboratory-confirmed 2009 A/H1N1v virus infection from August 1^st^ through December 31^st^, 2009 in metropolitan France to conduct a more comprehensive epidemiological study.

Here, we report the main demographic characteristics, maternal clinical course, treatment, and fetal and neonatal outcomes of 2009 A/H1N1v-infected pregnant or postpartum women with severe outcomes (admitted to the ICU), moderate outcomes (admitted to medical or obstetric wards), or mild outcomes (treated as outpatients) recorded in France during this period.

## Methods

### Implementation of the French registry

From August 1^st^ through December 31^st^, 2009, French physicians were invited to report all confirmed cases of 2009 influenza A/H1N1 in pregnant or postpartum women to the web-based French National Registry on Flu during Pregnancy. Women who demonstrated an onset of symptoms from 48 hours to 6 weeks after delivery were considered postpartum patients. A confirmed case was defined as a pregnant or postpartum women that presented with ILI and the diagnosis of 2009 influenza A/H1N1v by specific RT-PCR. Severe patients were defined as those who were admitted to an ICU, whereas moderate or mild patients were those who were admitted to medical or obstetric wards or treated as outpatients in the ambulatory setting, respectively.

The registry was based on the spontaneous reports of clinicians. French obstetricians, anesthesiologists, and intensivists were informed of the registry via nationwide e-mailing, specific intervention on the website of the scientific societies, and communication in several national meetings. A specific anonymous form was created on a specific and independent secured website. The report form was standardized and included data on demographics, past medical history, underlying medical diseases, clinical presentation and course (for the mother and the fetus), treatment and pregnancy outcome, if available.

We collected data on mothers' height and weight before pregnancy or on admission, any coexisting illnesses, gravidity, parity, estimated date of delivery, plurality, miscarriage (defined as fetal loss before 22 weeks of gestation), previous vaccination against seasonal or pandemic A/H1N1v influenza, and any medical or obstetric problems that developed during the current pregnancy. Obesity was defined as a pre-conception BMI over 30 kg/m^2^. We documented the date and time of delivery, the occurrence of labor (either spontaneous or induced), the indications for surgical delivery (such as maternal hypoxia or difficult ventilation, maternal hemodynamic instability, and/or fetal compromise), and the use of corticosteroids to induce fetal lung maturation. For each newborn, we recorded the method of birth (vaginal or surgical delivery), gestational age, birth weight, whether the newborns were live born or stillborn (fetal death ≥22 completed weeks of gestation), Apgar score at five minutes, admission and duration of admission to a neonatal intensive care unit (NICU) or special care nursery, neonatal infection with 2009 A/H1N1v influenza, the occurrence of any complications, and survival at the time of hospital discharge. Maternal and neonatal outcomes were last updated on March 31^st^, 2010. We made no assumptions regarding missing data. Missing data were excluded from the analysis, and all proportions were calculated as percentages of the patients with available data.

Regarding the most severe outcomes, REVA provided the missing data or files to be as exhaustive as possible, at least for this group of patients. From June 1^st^ through December 29^th^, 2009, 1015 hospitalizations for severe outcomes and 198 deaths related to suspected- or RT-PCR-confirmed 2009 H1N1 influenza were identified through enhanced surveillance in France by the InVS. Among these severe outcomes and deaths, 51 were recorded as pregnant or postpartum women [13].

Patients included in the R3G database were divided into three groups depending on the severity of the disease and the level of care: outpatient management for mild disease without other medical or social risk factors; hospitalization in obstetric or medical wards for moderate disease; and hospitalization in the ICU for severe disease.

The present study was an epidemiological study conducted under the aegis of four French medical societies (SFAR, CNGOF, SRLF, SPILF). All anonymous files concerning pregnant women with H1N1 pandemic flu were registered via the national secured website declared to and allowed by the CNIL, which is our National Committee on Informatics and Liberty. Data collection was conducted as part of an urgent public health response and was deemed exempt from review by an institutional review board. The data were collected from all regions of France, and therefore, no informed consent could be obtained from the patients.

### Statistical analysis

Continuous variables are expressed as means ± SD or medians [min-max] as indicated, and categorical variables are expressed as percentages. A comparison of the quantitative variables or proportions across groups was performed among the 3 groups using ANOVA with post-hoc tests or the chi-square or Fisher's exact test as appropriate. The odds ratio for developing a severe outcome and the 95% confidence intervals (CIs) were calculated using a conditional forward stepwise logistic regression. Only the variables that were significant at a limit of 5% in the univariate analysis were included in the multivariate model. A value of p<0.05 was considered significant.

## Results

**Figure 1:**
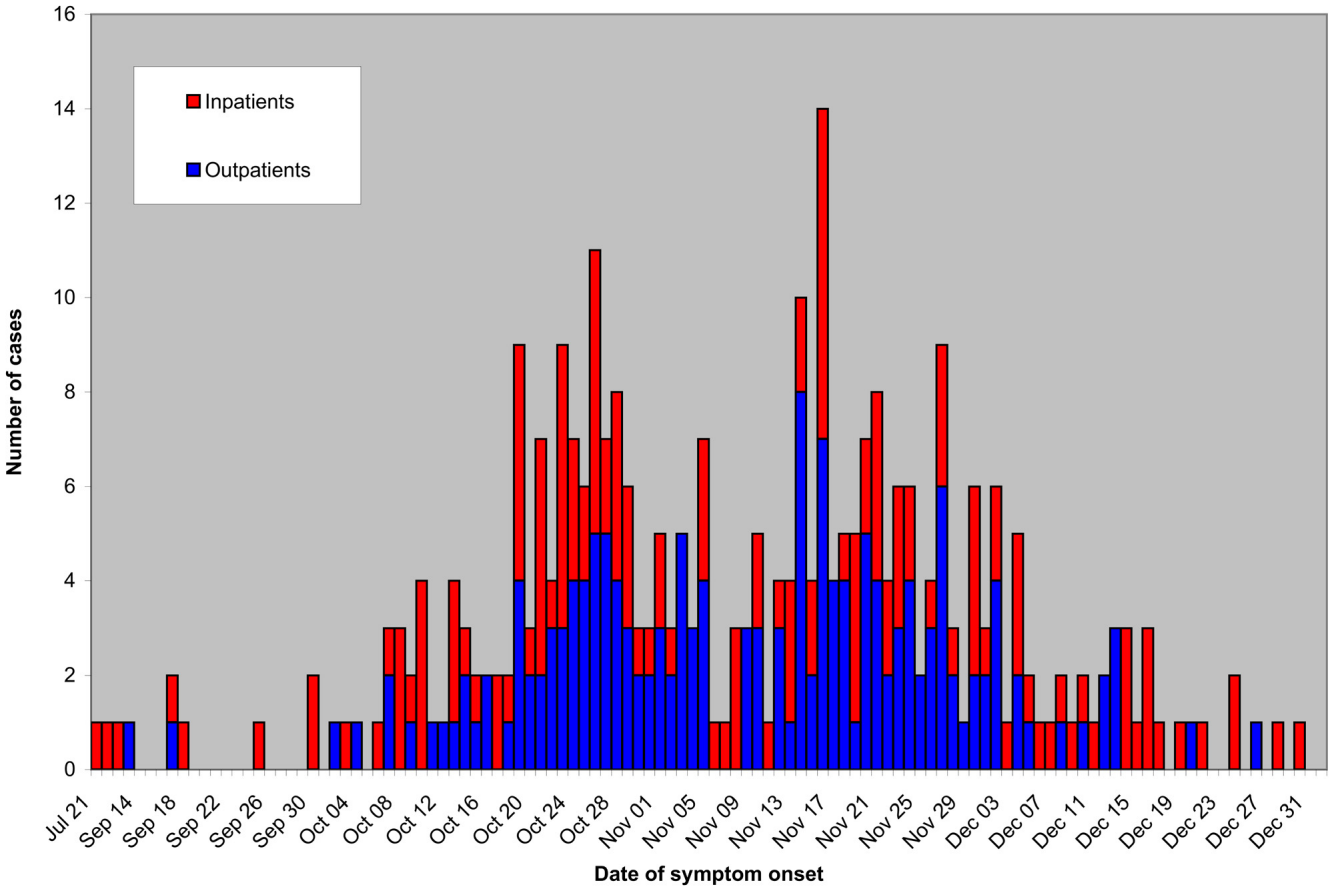
Temporal repartition of laboratory-confirmed 2009 A/H1N1v infection among pregnant women in the French registry according to patient status.

From August 1^st^ through December 31^st^, 2009, 315 pregnant or postpartum women with confirmed 2009 A/H1N1v influenza were included in the R3G database, and subsequent data were collected by the French web-based registry. Among these patients, 164 (52%) were outpatients, 111 (35%) were hospitalized in obstetric or medical wards, and 40 (13%) were hospitalized in an ICU. Dates of symptom onset ranged from July 21^st^ to December 30^th^, 2009 (Fig. 1), but most of the cases occurred between the 42^nd^ and 49^th^ week of 2009.

**Figure 2:**
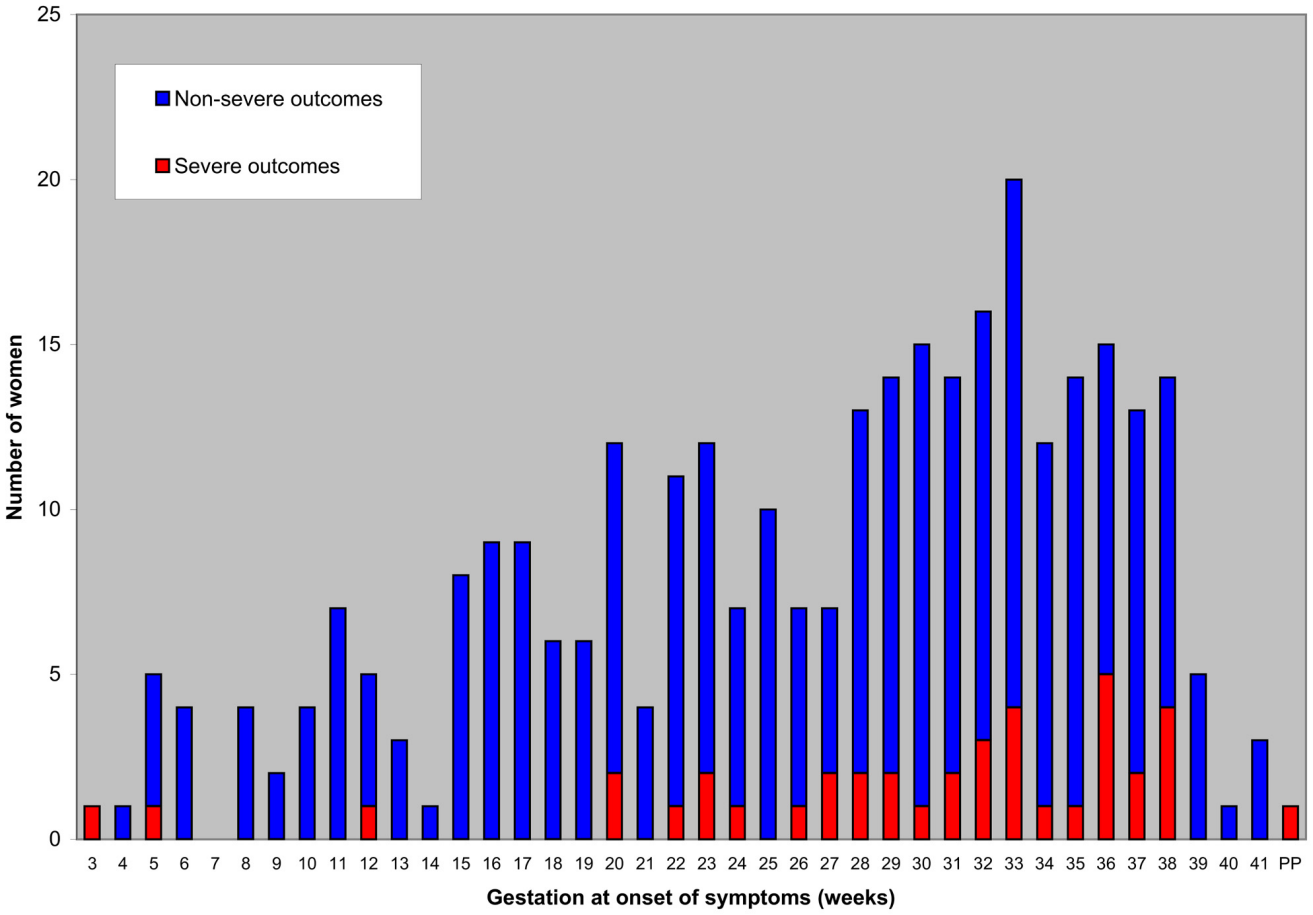
Gestational age at onset of symptoms according to the severity of symptoms.

Among the 164 outpatient women, 109 (66%) were in the first or second trimester of pregnancy, whereas 87 of the 111 hospitalized non-severe outcome women (78%) and 27 of the 40 ICU (most severe) patients (68%) were in the third trimester of pregnancy (Fig. 2, p<0.001). Only one ICU patient presented with influenza symptoms two days after an elective cesarean section following an uneventful pregnancy and was the single postpartum case. Five patients were twin pregnancies, and none of them had a severe outcome. Four women developed 2009 pandemic A/H1N1v influenza infection despite vaccination with the 2009 H1N1 monovalent vaccine. Notably, these four women had received the first injection of the vaccine less than 10 days before the onset of flu.

The main characteristics of the patients are summarized in Table 1. Coexisting illnesses were frequent, especially among ICU patients (23 of 40, 58%). In contrast, pregnancy *per se* was the only risk factor in 71% and 73% of the moderate and mild outcomes, respectively (p<0.001). Asthma (9%) and obesity (12%) were the most frequent co-morbidities among the 315 patients. Whereas there was no significant difference in the prevalence of obesity among the three groups, chronic respiratory disease was significantly more frequently reported among women admitted to the ICU (11 of 40, 28%) compared to the two other groups (p<0.001). Among the nine ICU patients with a history of asthma, four reported taking daily medication, which indicated severe chronic disease. The two other ICU patients with pulmonary disease had a restrictive syndrome (Steinert's disease and severe homozygous sickle cell anemia).

**Table 1:** Characteristics of pregnant or postpartum women with 2009 A/H1N1v influenza infection.

	ICU patients (severe) (n = 40)	Hospitalized non-severe patients (moderate) (n = 111)	Outpatients (mild) (n = 164)	p value
**Age, years**				
<20	5 (13)	3 (3)	2 (1)	
20–34	28 (70)	89 (80)	131 (80)	0.001
≥35	7 (18)	19 (17)	31 (19)	
Median age [min-max]	28 [17–45]	28 [15–43]	28 [14–45]	NS
**Pregnancy trimester**				
1^st^ trimester	3 (8)	0 (0)	33 (20)	
2^nd^ trimester	9 (23)	24 (22)	76 (46)	<0.001
3^rd^ trimester and postpartum	28 (70)	87 (78)	55 (34)	
**Chronic pre-existing disease**	23 (58)	32 (29)	44 (27)	<0.001
Obesity (BMI>30)	6	17	16	NS
Respiratory disease *	11	11	9	<0.001
Cardiac or vascular disease	3	1	5	NS
Metabolic disease other than diabetes**	1	2	6	NS
Diabetes except gestational diabetes	1	1	2	NS
Neurologic disease	1	2	5	NS
Renal disease	0	0	2	NS
Hepatic disease	1	0	1	NS
Hematologic disease	2	1	3	NS
Immunosuppression	1	1	1	NS
**Pregnancy-related disorders**	3 (8)	12 (11)	11 (7)	NS
Gestational diabetes	1	10	9	NS
Preeclampsia	1	0	2	NS
Others	2	2	0	NS
**Chronic medication (pre- or intra-pregnancy)**	13 (33)	11 (10)	18 (11)	<0.001
Corticosteroids	0	2	1	NS
Immunosuppression	0	0	1	NS
Others ***	13	9	17	<0.001

Values: N (%) unless stated otherwise. NS: non-significant.

*includes asthma, restrictive syndrome.

**includes thyroid and adrenal insufficiency.

***includes bronchodilators, insulin, salicylic acid, fractionated heparin, L-thyroxin, antihypertensive agents.

The most common symptoms of flu observed in this population were fever (86%), cough (84%), muscle aches (54%), headache (36%), and nausea/vomiting (10%), as shown in Table 2. Shortness of breath (22%) was more frequently reported among severe outcome ICU patients (p<0.001).

**Table 2:** Influenza symptoms, clinical course, and treatments of pregnant or postpartum women with 2009 A/H1N1v influenza.

	ICU patients (severe) (n = 40)	Hospitalized non severe patients (moderate) (n = 111)	Outpatients (mild) (n = 164)	p value
**Symptoms** *				
Hyperthermia >37.8°C	34 (85)	97 (89)	131 (85)	NS
Cough	28 (70)	93 (85)	134 (86)	0.04
Muscle aches	14 (35)	56 (51)	94 (61)	0.01
Headache	5 (13)	44 (40)	61 (39)	0.004
Rhinorrhea	3 (8)	34 (31)	51 (33)	0.006
Sore throat	2 (5)	14 (13)	19 (12)	NS
Shortness of breath	31 (78)	22 (20)	14 (9)	<0.001
Vomiting	6 (15)	14 (13)	10 (6)	NS
Diarrhea	1 (3)	1 (1)	3 (2)	NS
**Antiviral treatment**	40 (100)	109 (98)	150 (91)	0.01
≤2 days	18 (45)	87 (80)	132 (88)	
3-5 days	10 (25)	16 (15)	13 (9)	<0.001
>5 days	12 (30)	6 (6)	5 (3)	
**Initial antibiotic therapy**	32 (80)	75 (68)	65 (40)	<0.001
**Hospitalization ward**				
Obstetric ward	NA	94 (85)	NA	-
Medical ward	NA	17 (15)	NA	-
**Specific obstetric care** * **^,^** **	23/39 (59)	11/109 (10)	0/155 (0)	<0.001
Corticosteroids (fetal pulmonary maturation)	10 (26)	4 (4)	0 (0)	<0.001
Tocolysis	0 (0)	8 (7)	0 (0)	0.005
Artificial induction of labor	1 (3)	1 (1)	0 (0)	NS
Cesarean section	17 (44)	1 (1)	0 (0)	<0.001
**Fetal consequences** * **^,^** **	14/39 (36)	4/109 (4)	0/155 (0)	<0.001
Prematurity (<37 weeks of gestation)	13 (33)	2 (2)	0 (0)	<0.001
Miscarriage	0 (0)	1 (1)	0 (0)	NS
Stillbirth	1 (3)	1 (1)	0 (0)	NS

Values: N (%). NA: not applicable, NS: non-significant.

*Some data were only available for 109 non-severe hospitalized patients and 155 outpatients on March 31^st^, 2010.

**Information about obstetric care or fetal consequences is directly related to H1N1 illness. Obstetric care and fetal consequences were registered for only the 39 ICU-hospitalized women with a prepartum onset of influenza symptoms and excluding the only post-partum case in the registry.

All patients who presented with severe outcomes received antiviral treatment (oseltamivir alone or in combination with zanamivir in two patients) (Table 2). However, the delay from symptom onset to the initiation of antiviral treatment was more than 48 hours in 22 of the 40 (55%) ICU patients (median time 3 days [min-max 0-18]). In contrast, moderate and mild outcomes demonstrated a shorter duration to the initiation of antiviral treatment as compared to severe outcomes: 80 and 88% of moderate and mild outcomes, respectively, were treated less than 48 hours after onset of the illness (p<0.001).

Antibiotic therapy, mainly amoxicillin or cephalosporin combined with spiramycin, was often initially co-prescribed with the antiviral treatment in hospitalized patients (in 80% and 68% of cases of severe or moderate disease, respectively).

Among moderate outcomes, 94 (85%) were observed and treated in obstetric wards and 17 (15%) in medical wards. The most common reason for admission was ILI. Sixteen hospitalized women in the obstetric ward were admitted only for monitoring in the absence of severe influenza symptoms or pregnancy complications, mainly during the third trimester of pregnancy. Other patients were admitted for containment (n = 25), especially during the initial phase of the outbreak, pregnancy complications (n = 21), or decompensation of an underlying disease (n = 4).

Although none of the 164 outpatients required specific obstetric management, 11 of the 111 hospitalized non-severe (10%) and 23 of the 39 pre-partum severe patients (59%) required specific obstetric care, as shown in Table 2. Among the hospitalized non-severe patients, this specific obstetric care was mainly the treatment of preterm labor (8 of the 111 patients), whereas in the severe patients, it was mainly a cesarean section (17 of the 39 prepartum severe patients). Most of these 17 cesarean sections were conducted for maternal hypoxemia or a worsening condition. These cesarean sections induced preterm birth in only 13 patients: 1 at less than 29 weeks GA, 4 between 29 and 31 weeks GA, and 8 between 32 and 36 weeks GA. In contrast, there was no short-term fetal impact of the flu pandemic in the fetuses of 260 of 264 pregnant women with moderate or mild outcomes of the disease (98%) for whom data are available.

Forty women (13%) were hospitalized in the ICU. The mean SAPS II and SOFA scores at admission were 28±19 and 4±3, respectively. These patients were slightly younger (p = 0.001) than the non-severe patients. Ten (25%) had delivered prior to ICU admission. Most of the women (95%) were admitted to the ICU because of a respiratory failure, as shown in Table 3. At the time of admission, 32 of 40 women had pneumonia with an abnormal chest radiography or chest computed tomography (80%). Ten women (25%) presented with a documented secondary pulmonary infection during the hospital stay (*Streptococcus sp., Haemophilus influenza*, *Escherichia coli*, *Pseudomonas aeruginosa*, *Aspergillus fumigatus*, cytomegalovirus), and most of them were ventilator-associated pneumonias. Only four ICU patients had a documented bacterial co-infection at the time of admission (*Streptococcus sp.* in one woman and *Streptococcus pneumoniae* in three women). Cardiac dysfunction was observed in 17 patients (43%), either associated with respiratory failure in 16 patients or as a myocarditis without respiratory failure in the other patient. Twenty patients (50%) required mechanical ventilation for a median time of 13 days [2–55]. Among the twenty patients who required mechanical ventilation, eleven patients also required extracorporeal membrane oxygenation (ECMO) for a median duration of 8 days [4–38], among which nine survived. Five patients (13%) required non-invasive ventilation, and 15 (38%) required only mask or nasal oxygenation. The median ICU length of stay was 10 days [2–80]. Other ICU treatments are detailed in Table 3.

**Table 3:** ICU-hospitalized severe patients (n = 40).

**Post-partum admission to the ICU**	10 (25)
**Median time from symptom onset to ICU admission – days [min-max]**	3 [0–17]
**ICU indication(s):**	
Respiratory failure †	38 (95)
Cardiac failure	9 (23)
Neurologic failure	0 (0)
Renal failure ‡	1 (3)
Decompensation of an underlying pathology ¥	3 (8)
Other *	1 (3)
**Median SAPS II score on admission [min-max]**	22 [6–74]
**Median admission SOFA score [min-max]**	2 [0–13]
**Pathological admission pulmonary imaging ** ○	32 (80)
**Documented secondary pulmonary infection** ¶	10 (25)
**Ultimate respiratory failure:**	
None	1 (3)
Moderate hypoxemia	15 (38)
Acute lung injury (200<PaO2/FiO2<300)	5 (13)
Acute respiratory distress syndrome (PaO2/FiO2<200)	19 (48)
**Cardiac failure during hospitalization in the ICU ** ◊	17 (43)
**Neurologic failure during hospitalization in the ICU** ††	5 (13)
**Renal failure during hospitalization in the ICU**	6 (15)
**Ultimate respiratory care:**	
Oxygen therapy	15 (38)
Non-invasive ventilation	5 (13)
Mechanical ventilation (with or without ECMO)	20 (50)
ECMO	11 (28)
Median duration of ECMO – days [min-max]	8 [4–38]
**Other treatments:**	
Corticosteroids	12 (30)
Catecholamines	14 (35)
Renal replacement therapy	5 (13)
Transfusion	12 (30)
Other **	1 (3)
**Median length of ventilation, days [min-max]**	13 [2–55]
**Median ICU length of stay, days [min-max]**	10 [2–80]
**Death**	3 (8)

Values: N (%) unless stated otherwise.

† Respiratory failure included moderate hypoxemia (in fourteen cases), acute lung injury with 200<PaO2/FiO2<300 (in eight cases), acute respiratory distress syndrome (in thirteen cases), severe dyspnea without hypoxemia (in one case), and asthma crisis (in two cases).

‡ Thrombotic microangiopathy.

¥ Homozygous sickle cell anemia and vaso-occlusive crisis with acute chest syndrome (in one case), asthma (in one case), mitral stenosis and pulmonary edema (in one case).

*Other included postpartum hemorrhage.

○ Pathological pulmonary imaging including interstitial syndrome (in nineteen cases), alveolar syndrome (in twenty-eight cases), and pleural effusion (in two cases).

¶ Secondary pulmonary infection included *Streptococcus pneumoniae* (in three cases) and *Streptococcus sp.*, *E. coli*, *Pseudomonas*, *Haemophilus*, *Klebsiella pneumoniae*, *Cytomegalovirus*, and *Aspergillus fumigatus* infections (in one case each).

◊ Cardiac failure included cardiac dysfunction (in eight cases), vasoplegia (in eight cases), short cardiac arrest (in two cases), and pericarditis (in four cases).

†† Neurologic failure included ischemic cerebro-vascular disease (in one case), critical illness neuromyopathy (in two cases), hallucinations (in one case), and psychomotor agitation (in one case).

**Other included immunoglobulins for the treatment of thrombotic microangiopathy.

Three women died (8%). Two of them were in the third trimester of pregnancy at symptom onset, and the third was in the second trimester. All of these patients had coexisting illnesses: obesity in one (body mass index: 31 kg/m^2^), severe thrombotic microangiopathy in another, and cardiac valvular disease (mitral stenosis and aortic regurgitation) in the last patient. Only one of them received antiviral medication within 48 hours after symptom onset. The other two received oseltamivir at 8 and 9 days after first symptom onset, likely due to poor access to care. Refractory acute respiratory distress syndrome (ARDS, PaO2/FiO2<200) was diagnosed in two of these three patients for whom ECMO was unsuccessful; the third patient with acute lung injury (ALI, 200<PaO2/FiO2<300) received only protective mechanical ventilation but died due to massive cerebral ischemia in the context of thrombotic microangiopathy.

The multivariate analysis of risk factors for developing a severe outcome is shown in Table 4. We found a strong association between the development of a severe outcome from flu and both co-existing illnesses (adjusted odds ratio [OR], 5.1; 95% confidence interval [CI], 2.2–11.8; p<0.001) and a delay in oseltamivir treatment after the onset of symptoms (>3 or 5 days) (adjusted OR, 4.8; 95% CI, 1.9–12.1, p = 0.001, and 61.2; 95% CI, 14.4–261.3, p<0.001, respectively).

**Table 4:** Impact of coexisting illnesses and the timing of antiviral treatment on admission to an intensive care unit.

	Adjusted OR	95% CI	p value
**Coexisting illness**	5.11	2.22–11.78	<0.001
**Delay of treatment <3 days** after symptom onset	Reference	-	-
**Delay of treatment 3–5 days** after symptom onset	4.78	1.89–12.09	0.001
**Delay of treatment >5 days** after symptom onset	61.24	14.35–261.25	<0.001

OR, odds ratio; CI, confidence interval.

On March 31^st^, 2009, pregnancy outcomes were known for 146 of the 231 patients with an estimated delivery date prior to March 31^st^ (63%). Most of the outpatients and non-ICU hospitalized women delivered vaginally (85% and 73%, respectively), as shown in Table 5. Among the 45 outpatient women who had delivered, only three of their neonates required immediate resuscitation. Moreover, only one of the 45 neonates of the outpatient women for whom data were available was admitted to the neonatal ICU.

**Table 5:** Perinatal outcome.

	ICU patients (severe) (n = 33)	Hospitalized non-severe patients (moderate) (n = 66)	Outpatients (mild) (n = 47)	p value
**A. PREGNANCY OUTCOME**				
**Vaginal delivery (live birth)**	11 (33)	48 (73)	40 (85)	<0.001
**Cesarean delivery (live birth)**	20 (61)	16 (24)	5 (11)	<0.001
**Intra-uterine fetal death**	1 (3)	1 (2)	0 (0)	NS
*term – weeks of gestation*	21	29	-	
**Miscarriage**	1 (3)	1 (2)	1 (2)	NS
*term – weeks of gestation*	-	23	18	
**Termination of pregnancy**	0 (0)	0 (0)	1 (2)	NS
*median term – weeks of gestation [range]*	-	-	10	
**B. NEONATAL OUTCOME** *				
**Median gestational age – weeks [min-max]**	37 [27–41]	38 [24–41]	40 [29–42]	<0.001
*< 29 weeks of gestation*	2/31 (6)	1/64 (2)	0/45 (0)	
*29–31 weeks of gestation*	3/31 (10)	0/64 (0)	1/45 (2)	
*32–36 weeks of gestation*	9/31 (29)	8/64 (12)	2/45 (4)	
*≥37 weeks of gestation*	17/31 (55)	55/64 (86)	42/45 (93)	
**Median birth weight - grams [min-max]**	2780 [1215–4110]	3270 [550–4670]	3350 [1520–4380]	0.01
*<1500*	2/31 (6)	1/63 (2)	0 (0)	
*1500–2499*	12/31 (39)	6/63 (10)	1/44 (2)	
*≥2500*	17/31 (55)	56/63 (89)	43/44(98)	
**Newborn resuscitation in the L&D unit**	15/31 (48)†,¥	8/61 (13)	3/45 (7)	<0.001
**Admission to neonatal intensive care unit**	14/31 (45)	4/61 (7)	1/45 (2)	<0.001
**Neonatal death (in the L&D unit or in the neonatal intensive care unit)**	1/31 (3)	1/61 (2)	0/45 (0)	NS

Values: N (%) unless stated otherwise.

L&D: labor and delivery.

NA: not applicable. NS: non-significant.

*vaginal or cesarean delivery.

† intubation in 10 cases, resuscitation in 2 cases.

¥ Apgar <4 at five minutes in 3 cases.

In contrast, most of the severe outcome ICU-admitted women were delivered by cesarean section (20 of 33) with a median gestational age of 33 weeks. Most of the cesarean deliveries (17 of 20) were directly related to influenza illness. These cesarean sections were mainly emergent procedures for both fetal indications and critical maternal hypoxemia (9 of 17). In two patients, the cesarean section was performed only for fetal indications (non-reassuring fetal heart rate), and in six patients, it was performed only to improve maternal oxygenation. In two patients, the cesarean section was performed in the ICU because of severe refractory hypoxemia that was incompatible with transfer to an operating room.

Half of the neonates of women admitted to the ICU for whom data were available were admitted to the NICU, as shown in Table 5. Among the three maternal deaths, one had delivered a healthy full-term neonate at term by cesarean section (indicated because of thrombotic microangiopathy) before the ICU admission, one experienced an intra-uterine fetal death at 21 weeks of gestation, and the third delivered at 34 weeks of gestation by cesarean section to improve maternal oxygenation and for fetus salvation. This infant was admitted to the neonatal ICU due to prematurity and low Apgar scores (5 at 1 minute, 7 at 5 minutes) and had survived at the time of NICU discharge. Data collection for definitive neonatal morbidity and mortality is still in progress, but to date, no cases of A/H1N1v influenza have been reported in neonates.

## Discussion

These are the first reported results of the largest cohort of pregnant or postpartum women with confirmed 2009 A/H1N1v influenza, including severe, moderate, and mild outcomes of the disease, in a European country involved in the flu outbreak at the end of 2009.

Several comments are raised by this study. First, the present results confirm the data reported for past pandemics [1]–[4], which indicate an increased risk for pregnant and/or postpartum woman in developing a severe outcome of pneumonia and/or ARDS during pandemic flu. Earlier in the spring and summer of 2009, similar observations from the USA, Mexico, Canada, Australia, and New Zealand were published, with pregnant or postpartum patients accounting for 8 to 10% of H1N1-related critically ill patients and 2 to 4% of deaths, whereas child-bearing-aged women in these countries represent roughly 1% of the population [8], [14]–[16]. Our current data confirm that in France, pregnancy again constituted a significant risk factor for severe illness during the 2009 pandemic flu. Interestingly, Hanslik et al. measured the risk for admission into an intensive care unit for a pregnant woman and found an odds ratio of 5 [95% CI 4.0–6.9] [17].

Second, and in contrast with the above-mentioned experience from the 2009 pandemic influenza in other countries [7], [9], [18]–[20], pregnancy did not seem to significantly increase mortality in our country. The R3G registry indeed recorded only three deaths in France in pregnant or postpartum patients, and this finding was confirmed by the InVS. In the work by Hanslik, heart failure, obesity, and diabetes, but not pregnancy, were significantly associated with death in France [17]. Interestingly, very recent data from other countries in Asia, although comprising a smaller series and from a single center, seemed to demonstrate the same trend [21]. It is tempting to hypothesize that such an observation could be partially to the following factors: (i) first, patient characteristics (less obesity or coexisting diseases in our population and in our patients in comparison with other series). Comparisons of the incidence of co-existing illness between our cohort and the American and Australian ones [18]–[20] revealed a lower incidence of obesity (12% versus 20 to 42%) and asthma (9% versus 12 to 20%); (ii) second, early diagnosis and a shorter delay until the initiation of antiviral treatment in our study, retrospectively validating the French guidelines; (iii) third, very good compliance of the physicians and patients; (iv) fourth, an increased awareness of the specific severity of pandemic flu during pregnancy arising from international experience.

When comparing non-severe with severe outcomes of pandemic flu in French pregnant or postpartum women in the registry, the main results were as follows: i) a lower incidence of coexisting illnesses (28% compared with 58%), mainly respiratory disease, and ii) a significantly shorter delay between the onset of disease and initiation of oseltamivir treatment. Evidence for the benefit of oseltamivir in preventing influenza complications remains insufficient to date [22], yet suggested by our series and those from the USA or Australia [18]–[20]. In April 2010, the Centers for Disease Control and Prevention (CDC) reported on 788 pregnant women in the United States with 2009 A/H1N1v influenza with symptom onset from April through August 2009 [23]. Among these patients, early antiviral treatment appeared to be associated with fewer admissions to an ICU and fewer deaths, although this result was not adjusted for coexisting illnesses or the pregnancy trimester. Our series confirmed that a delay in oseltamivir treatment after the onset of symptoms constituted an independent risk factor associated with the development of more severe outcomes of 2009 A/H1N1v influenza in pregnant women. We hypothesize that earlier treatment with oseltamivir may also reflect easier access to care and more informed physicians, which may have allowed for prompt recognition of the most severe patients, thus reducing subsequent severe morbidity or mortality due to better triage and care.

Finally, the obstetric and neonatal consequences of pandemic flu observed in France appeared to be rather limited. Consistent with the first reports from Victoria in Australia [19], New York [20], and California [9], the 2009 A/H1N1v influenza illness significantly increased the need for cesarean section delivery, mainly due to worsening maternal conditions. Cesarean section did not seem to worsen maternal conditions, because only one of the 17 women died with critical hypoxemia. Intensive management for maternal hypoxia, including extracorporeal membrane oxygenation, also led to better than expected outcomes when compared with the usual 30 to 35% mortality rate reported for ARDS. The NICU admission rate also increased, mainly because of preterm deliveries that comprised almost exclusively fetuses from the most severely ill pregnant women. In contrast, a worrisome observation from Australia and the USA reporting increased neonatal morbidity and mortality did not seem to occur in our cohort [19], [20]. Severe neonatal morbidity and mortality have remained extremely low (2 stillbirths and 2 neonatal deaths). These data are very important because they were collected from a large series of infants (146) delivered by mothers who had been treated with oseltamivir, thereby reinforcing very recent data on the safety of this antiviral drug during pregnancy [24].

One important limitation of this study was the lack of exhaustive documentation of non-severe outcomes of pregnant women who suffered from the 2009 pandemic flu in France. However, we did include 275 patients with non-severe outcomes of pandemic flu during pregnancy, which is the most important to our knowledge in Europe to date. One may argue that a comparison of mostly severe patients and some non-severe patients, who were registered based on the willingness of physicians, may be biased. Let us assume that the worst biases will cause the 275 non-severe outcomes in the registry to become less mild or moderate outcomes than were indeed the case in real life. Consequently, significant differences between severe and non-severe patients in the registry will likely be clinically significant when they are applied to the entire population.

In conclusion, the French experience of the pandemic 2009 A/H1N1v flu in pregnant women resulted in an increase in morbidity and ICU management of some patients, mainly at-risk patients who were likely diagnosed and/or treated too late. Maternal mortality has remained low and was lower than that observed in other comparable countries. Moreover, at least in France, the obstetric outcome and neonatal mortality did not appear to be seriously affected by the H1N1 outbreak. This result may be due to wider access to care, directive guidelines, good compliance from patients and physicians, and early diagnosis and treatment with oseltamivir.

**What This Paper Adds**

**What is already known on this topic**

- Pregnant women are at an increased risk for complications and mortality due to influenza.
- Intensive management for maternal hypoxia, including cesarean section and extracorporeal membrane oxygenation, can lead to better-than-expected outcomes.

**What this study adds**

- Lessons from international experience promoted national guidelines that recommend earlier treatment with oseltamivir and improved triage and care.
- This could have been responsible for the lower maternal and neonatal mortality and morbidity observed in France when compared with the USA and Australasia.
